# The effects of mHealth interventions on improving institutional delivery and uptake of postnatal care services in low-and lower-middle-income countries: a systematic review and meta-analysis

**DOI:** 10.1186/s12913-023-09581-7

**Published:** 2023-06-09

**Authors:** Reta Tsegaye Gayesa, Fei Wan Ngai, Yao Jie Xie

**Affiliations:** 1 School of Nursing, The Hong Kong Polytechnic University, Hong Kong S.A.R, China; 2 Institute of Health Sciences, Wollega University, Nekemte, Ethiopia

**Keywords:** mHealth, Women, Meta-analysis, Postnatal care

## Abstract

**Background:**

Maternal mortality due to pregnancy, childbirth and postpartum is a global challenge. Particularly, in low-and lower-income countries, the outcomes of these complications are quite substantial. In recent years, studies exploring the effect of mobile health on the improvement of maternal health are increasing. However, the effect of this intervention on the improvement of institutional delivery and postnatal care utilization was not well analyzed systematically, particularly in low and lower-middle-income countries.

**Objective:**

The main aim of this review was to assess the effect of mobile heath (mHealth) interventions on improving institutional delivery, postnatal care service uptake, knowledge of obstetric danger signs, and exclusive breastfeeding among women of low and lower-middle-income countries.

**Methods:**

Common electronic databases like PubMed, EMBASE, the Web of Science, Medline, CINAHL, Cochrane library, Google scholar, and gray literature search engines like Google were used to search relevant articles. Articles that used interventional study designs and were conducted in low and lower-middle-income countries were included. Sixteen articles were included in the final systematic review and meta-analysis. Cochrane’s risk of bias tool was used to assess the quality of included articles.

**Results:**

The overall outcome of the systematic review and meta-analysis showed that MHealth intervention has a positive significant effect in improving the institutional delivery (OR = 2.21 (95%CI: 1.69–2.89), postnatal care utilization (OR = 4.13 (95%CI: 1.90–8.97), and exclusive breastfeeding (OR = 2.25, (95%CI: 1.46–3.46). The intervention has also shown a positive effect in increasing the knowledge of obstetric danger signs. The subgroup analysis based on the intervention characteristics showed that there was no significant difference between the intervention and control groups based on the intervention characteristics for institutional delivery (*P* = 0.18) and postnatal care utilizations (*P* = 0.73).

**Conclusions:**

The study has found out that mHealth intervention has a significant effect on improving facility delivery, postnatal care utilization, rate of exclusive breastfeeding, and knowledge of danger signs. There were also findings that reported contrary to the overall outcome which necessitates conducting further studies to enhance the generalizability of the effect of mHealth interventions on these outcomes.

## Background

According to World Health Organization report, maternal death due to complications related to pregnancy, childbirth, and postpartum is a global challenge that disproportionately affects countries of low-income settings [1]. This report also indicated that by the end of 2017, 86% of global maternal deaths have occurred in Sub-Saharan Africa and Southern Asia. Many maternal and neonatal health complications are caused by a lack of access to high-quality maternal care, such as skilled birth attendance, facility-based delivery, and postnatal care services [2]. In low- and middle-income countries, the primary strategy for reducing maternal and neonatal mortality has been to increase the rate of deliveries in health facilities [3].

In the first 42 days after giving birth, especially in the first week, postpartum is when most maternal and baby deaths occur [4]. This evidence emphasizes why facility-based delivery and postnatal care provision are so important to avert the burden of maternal and neonatal complications. According to the World health organization (WHO), postnatal care is a neglected service along the postnatal care continuum [5]. By 2030, countries are anticipated to reduce the maternal mortality to meet one of the sustainable development goals (SDGs) key agenda about maternal and child health. Particularly, the SDG 3 is targeted in: “lowering maternal mortality rates (MMR) worldwide to less than 70 per 100,000 live births, with no country having MMRs that are more than twice the global average” [1].

In recent years, with the advancement of technology, the use of mobile health in health care is increasing rapidly and is anticipated to enhance maternal and child health care services [6]. Due to its accessibility and cost-effectiveness, this technology is holding considerable promise in the healthcare system. Mobile health (mHealth) is a way of communicating using wireless devices to enhance healthcare services for illness prevention, disease treatment, and health promotion [7]. It positively affects the health care system by improving access to quality health care and reducing the cost of health services. Smartphones, handheld devices, personal digital assistants (PDAs), and mobile phones with PDA features are all examples of PDAs and are the most often used tools or technology in mobile health [8].

The number of studies conducted to determine the impact of mHealth on maternal and child health is also growing. Though few existing reviews focused on effect of mHealth on health care cost outcomes, there were also few literatures on maternal health care services [8, 9]. Existing evidences however showed inconclusive findings. A systematic review conducted by Chen et al. (2018) revealed that nearly half (43%) of included primary studies had shown negative or unclear results on the effect of mHealth interventions on maternal and child healthcare [9]. On the other hand, a meta-analysis aimed to identify effect of mhealth on antenatal care visits and skilled delivery showed promising positive effect mHealth interventions despite significant heterogeneity among the studies [10]. Other existing primary studies also showed varying effects of mhealth on different maternal health services utilizations [11–14]. To the best of our knowledge, however, past research did not assess the effect of mobile health on additional maternal care outcomes and related postnatal care practices, such as exclusive breastfeeding and level of awareness of obstetric danger signs. Thus, this review and meta-analysis was aimed to assess the effect of mHealth interventions on improving institutional delivery, postnatal care service uptake, knowledge of obstetric danger signs, and exclusive breastfeeding among women of low and lower-middle-income countries.

## Methods

### Search strategy

The population, intervention, control, and outcome (PICO) framework were used to formulate a question for this systematic review. Accordingly, population refers to the pregnant or laboring mother and postnatal women; intervention refers to a mobile educational message, SMS/voice reminder message, or combination of both reminder and educational message; control refers to the routine maternal care provided by a health care professional, and outcome refers to the level of utilization of postnatal care (measured as complete and incomplete utilization), level of institutional delivery or skilled birth attendance, and level of exclusive breastfeeding or self-efficacy of breastfeeding. The study protocol was also registered on prospective register of systematic reviews (PROSPERO) (ID = CRD42022366738).

Only published articles until October, 2022 were searched from common electronic databases like PubMed, EMBASE, CINAHL, the Web of Science, Medline, Cochrane library, and Google scholar and gray literature search engine like Google. Search terms were also aligned with the PICO framework. These search terms include; mHealth OR mobile health OR sms OR mobile phone* OR mobile telephone* OR cellphon* OR cell phon* OR text messag* OR short message service* OR ehealth OR e-health OR smartphone* OR smart phone* OR mobile device* OR electronic device* OR phone intervention* OR telephon* intervention* OR online OR mobile app OR reminder OR reminder messag*

The search terms for population includes mother* OR families* OR parent* OR women OR woman OR pregnant*

The search term for outcome was postnatal care OR post-natal care OR maternal care OR maternity care OR postpartum care OR "Postnatal Care"[Mesh] OR institutional delivery OR facility delivery, knowledge, "Health Knowledge, Attitudes, Practice"[Mesh] OR health care seeking OR breastfeeding* OR exclusive breast feeding OR "Breast Feeding"[Mesh] OR self-efficacy OR utilization OR uptake OR behavior OR skill*

The search results were then limited to studies published in English, and original articles of randomized controlled studies in low and lower-middle income countries.

### Inclusion and exclusion criteria

**Fig. 1:**
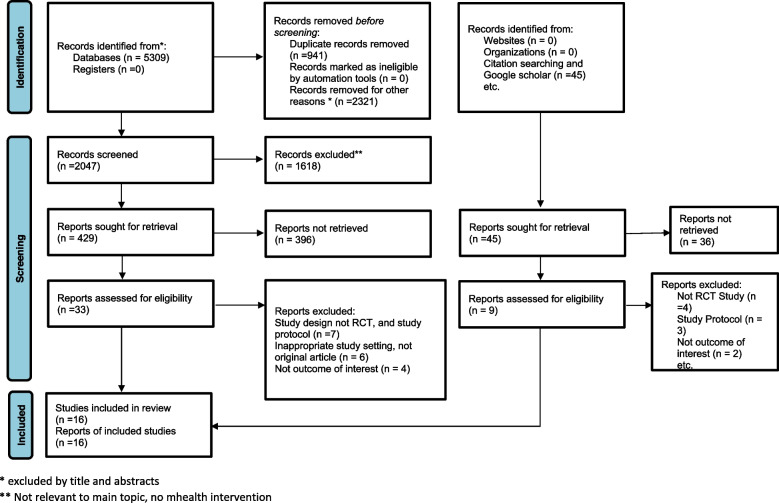
PRISMA flow diagram of selected studies for systematic review and meta-analysis of effect of mhealth interventions on institutional delivery and PNC uptake in low and lower-middle income countries

Articles conducted in low and lower-middle-income countries published in English till October 2022 were included. Low-income economies are defined as having a GNP per capita of $1,085 or less in 2021; lower-middle-income economies have a GNP per capita between $1,086 and $4,255 [15]. This systematic review and meta-analysis only included articles published by interventional study designs like true or quasi-randomized controlled trials and interventional designs with historical cohort. Articles should be conducted on pregnant mothers or postpartum women to be included. Articles should also use mHealth as an intervention and usual (routine) care as a control. Included studies should also report at least one outcome from the rate of institutional delivery, postnatal care uptake, exclusive breastfeeding, and knowledge of obstetric danger signs during pregnancy or postpartum. Study protocols and articles published in other than the English language were excluded. Moreover, this systematic review followed Preferred Reporting Items for Systematic Reviews and Meta-Analysis (PRISMA) flow diagram to indicate the detailed procedure of flow of the review (Fig. 1) [16].

## Study outcome

### Quality assessment of articles

Critical appraisal of the included articles was done by using the Cochrane risk bias tools for RCT and quasi-randomized studies [17]. The tool consists of seven components of bias assessment. These are selection bias comprising random sequence generation and allocation concealment, reporting bias, performance bias, detection bias, attrition bias and other bias. The bias was independently assessed by the principal author and involved co-authors.

### Data abstraction

The following types of data were extracted; 1) basic information about the study, such as the author, publication year, and the country or study setting, 2) the target population, 3) the type of mHealth intervention, and the frequency and duration of the mHealth intervention; 4) the study design and the number of participants given the mHealth interventions (sample size); 5) the primary and secondary outcome and 6) the summary results of the study (Table 1).

**Table 1:** Characteristics of included studies and their summary of results

Authors (year)	Study setting (country)	Study population and design	Sample size intervention and control	Intervention type, frequency and duration	Control Group	Primary and secondary Outcomes	Summary of Main findings
Kebede et al. 2019 [9]	Ethiopia	Postnatal women, RCT	I = 350; C = 350	SMS or voice call reminder 48 and 24 h reminders ahead of each PNC visit	Usual care	PNC adherence	Sms reminder enhances adherence to PNC utilization
Omole et al. 2018 [10]	Nigeria	Pregnant women, RCT, four hospitals	I = 260; C = 248	Pregnancy related text messages until 6 weeks after child birth	General health message	ANC and Institutional delivery rates	SMS message increased ANC visit and facility delivery rate (29% vs 13% among intervention and control respectively with effect size of 14%)
Olajubu et al. 2020 [11]	Nigeria	Pregnant women, Quasi experimental	I = 190; C = 190	SMS reminder and educational message (before birth: from Week 35 until birth: 2 messages per week) (After birth: Week 1: 3 messages (days 2,3,5) Weeks 2–6: 2 messages per week (i.e., between day 1 to 41) Reminder for Postnatal visits: day 1, 2, 9 and 41 Content of educational message: PNC, BF, self-care, danger signs, cord care, immunization	Usual care	PNC service utilization	SMS educational message improves PNC utilization (30.9% vs. 3.7%, AOR: 10.869, 95% CI: 4.479–26.374)
Shiferaw et al. 2016 [12]	Ethiopia	Pregnant women Nonrandomized CT	I = 613; C = 568	reminders for scheduled visits 7 days and 3 days before schedule visit for ANC and PNC and educational messages on dangers signs	Continued with Usual care	ANC attendance, institutional delivery, and PNC service utilization	Improved ANC, institutional delivery and PNC by positive behavioral change
Fedha et al. 2014 [13]	Kenya	Pregnant women, prospective RCT	I = 191; C = 206	Reminder message every forty night of next visit to the clinic and given advice on pregnancy updates	Continued with Usual care	Proportion ANC use and facility delivery	Mhealth improved ANC and facility delivery uptake
Lund et al. 2012 [14]	Zanzibar	Pregnant women, Cluster RCT	I = 1311 C = 1239	Mobile message every 2 weeks in early pregnancy, and twice a week after 36 weeks	Usual care	Skilled delivery attendance	The intervention produced a significant increase in skilled delivery attendance amongst urban women (OR, 5.73; 95% CI: 1.51–21.81)
Atnafu et al. 2017 [15]	Ethiopia	Pregnant women, Community based RCT (2 intervention, 1 control)-Pre and post intervention	I = 2160 C = 1080	SMS message at 14, 24, 30, 36 weeks during pregnancy and at 6,10,14 weeks, and at 9 month for vaccination Contents were scheduled date of ANC visit, expected date of delivery, PNC, Immunization schedule and vaccine and contraceptive stock status	Usual care	Percentage of ANC visit and skilled delivery	Mhealth improved the percentage ANC uptake and delivery by skilled attendant
Jones et al. 2020 [16]	Kenya	Postnatal women, RCT, 4-arms (1 control and 3 intervention)	I1 = 222 I2 = 223 I3 = 229 C = 227	Messages covered danger signs, general postpartum topics, And family planning 4 messages per day on day 2, 3, 4 after discharge (PPC message -I1) Every 3 days after discharge from day 6–36 PNC message -I2) Every 3 days from day 30–45 (FP message-I3)	Usual care	knowledge, PNC care seeking behavior and family planning uptake	SMS message has positive effect on PNC seeking behavior, and improved family planning uptake and knowledge
Flax VL et al. 2017 [17]	Nigeria	Pregnant women, Cluster RCT	I = 196 C = 194	1) breastfeeding learning sessions 2) Cell phone text and voice messages**;** and 3) songs and dramas on group meeting. Cell phone text and voice messages was sent to a phone provided to each group of intervention contents: early initiation, and EBF messages	Usual group meeting, no message provided	Assessing early BF initiation and EBF	The odds of exclusive breastfeeding to 6 mo (OR: 2.4; 95% CI: 1.4, 4.0) and timely breastfeeding initiation (OR: 2.6; 95%CI: 1.6, 4.1) were increased in the intervention vs. control arm
Ungar JA et al. 2018 [18]	Kenya	Pregnant women, 3-arm individually randomized controlled trial	I1 = 93, I2 = 91, C = 94	Weekly SMS message tailored for maternal characteristics and pregnancy Or postpartum timing	Usual care	Facility delivery, EBF and contraceptive use	No significant difference on facility delivery but probability of EBF was higher in 1-way SMS arm at 10 and 16 weeks, and in 2-way SMS arm at 10, 16, and 24 weeks
Bangal et al. 2017 [19]	India	Pregnant women, Prospective RCT	I = 170 C = 15 SBA I = 185 C = 190	Mobile call and SMS reminder at regular interval	Routine antenatal and postnatal care	antenatal visits, percentage of institutional delivery and postnatal check- ups	Women in the intervention group had significantly higher number of antenatal visits, consumption of iron tablets, tetanus toxoid immunization, institutional deliveries and postnatal check-ups as compared to the control group
Ogaji et al. 2020 [20]	Nigeria	Postnatal women Prospective RCT	I = 67 C = 64	Reminding mothers of the benefits of EBF for their babies and receiving feedback if any challenges	received ‘usual care’	Rates and duration of exclusive breastfeeding (EBF	Mobile phone-based support had improved the rates and duration of EBF
Adam et al. 2021 [21]	South Africa	Pregnant women, cluster RCT	I = 423 C = 501	Video uploaded mobiles were given to mentor mothers. The video demonstrates information and techniques of EBF methods	Usual care	Primary outcome: EBF practices at 1 month and at 5 month Secondary outcome: early initiation of BF, complementary feeding and maternal knowledge	No statistically significant differences between the 2 study arms at 5 months for either of the primary outcomes: EBF in the last 24 h (RR 0.90, 95% CI 0.77 to 1.04, P = 0.152) or since birth (RR 0.78, 95% CI 0.78 to 1.08, P = 0.282; No significant knowledge difference at 5^th^ month but at 1^st^ month
Hmone et al. 2017 [22]	Myanmar	Pregnant women, RCT	I = 143 C = 137	short message service (SMS) on breastfeeding promotion	Only message related to pregnancy support not BF	Primary outcome: to assess the EBF rate at 1–6 month, 2ndry outcome: median duration of EBF and BF self-efficacy	The intervention significantly increased the EBF rate over the 6 months of follow-up (Relative Risk (RR), 1.54; 95% CI: 1.41–41.69; P < .001), at each monthly follow-up visit. Women from the intervention group had higher breastfeeding self- efficacy during the follow-ups
Adanikin et al. 2014 [23]	Nigeria	Postpartum women, historic control group interventional design	I = 1126 C = 971	SMS appointment reminder	Usual care	Attendance rates at postnatal clinics	Text reminders led to a 50% reduction in failure to attend to postnatal clinic appointments
Seyyedi et al. 2021 [24]	Iran	Postnatal women, RCT	I = 40 C = 40 mean self-efficacy scale	Smart-phone based education	Routine care	Breast feeding self-efficacy, KAP on BF	The smartphone-based app educational message had a significantly positive effect on breastfeeding self-efficacy and maternal KAP on BF

*I* Intervention group, *C* Control group

### Data analysis and synthesis

The data was analyzed by review manager 5 (RevMan version 5.4) for articles in which the outcomes were reported in the figures. For those articles from which figures were not extracted, we have discussed the overall outcome of the study with other pooled findings and relevant literatures. The heterogeneity of the studies was assessed by I^2^ test statistics. The value of I2 statistic was defined as no heterogeneity, moderate heterogeneity, and high heterogeneity at 25%, 50%, and 75% respectively [17]. The sensitivity analysis was conducted by leave-one out approach to identify the effect of single study influence on the overall study result. The random and fixed effect models were used based on the level of heterogeneity of included studies for all the required outcomes. The subgroup analysis was conducted based on the intervention characteristics.

### Ethical approval and consent to participate

The study did not require ethical approval and consent to participate because we used already published articles.

## Results

### Characteristics of included studies

Sixteen articles were finally included in this systematic review and meta-analysis. These studies were published between 2012 and 2022. The sample size in the primary articles ranged from 91 to 2160, and a total of 14,410 study subjects participated in the current systematic review and meta-analysis. Thirteen of the included studies were RCTs two articles were quasi-experimental studies and one study was interventional design with the historic control group. Included studies were conducted in Nigeria (5), Kenya (3), Ethiopia (3), India (1), South Africa (1), Myanmar (1), Iran (1), and Zanzibar (Tanzania) (1). Among eligible studies, three studies were excluded because they were study protocols [18–20], not done in Low-and lower-middle-income countries [21, 22], did not report the outcome of interest [6, 23, 24], and were not original articles [25–27].

### Intervention characteristics

Among the included studies, seven of them have addressed the effect of mHealth on institutional delivery [28–34] while six of them have addressed its effect on the uptake of postnatal care [11, 13, 14, 29, 34, 35]. Six of the included studies have addressed its effect on the level of exclusive breast feeding practices among the target population [12, 33, 36–39] while two articles examined the effect of mHealth intervention on postpartum knowledge of maternal and newborn care [12, 14]. Majority of studies enrolled the participants during late pregnancy while few others enrolled the participants immediately after delivery, varying based on the desired outcome. The duration of intervention also varies across studies based on the outcome needed. For facility delivery, the intervention had started as early as 14 weeks during pregnancy and as late as 35 weeks during pregnancy. For postnatal care outcome, the intervention started starting from 35 weeks of pregnancy in some studies and immediately after delivery in other studies. The intervention components were short message (SMS) or voice call reminders in four studies [11, 13, 32, 34], the specific educational message was used in nine studies [12, 14, 28, 31, 33, 36–39] and combined reminder and educational message were used in three studies [29, 30, 35]. The content of the message varies across studies but they were derived from Mobile alliance for maternal action (MAMA) message [40] and WHO recommendations for postnatal care services and other literature searches. The measurement points for facility delivery were during pregnancy (recruitment) and childbirth while the measurement points for postnatal care utilization were at day 1, day 3, day 10, and 6 weeks after childbirth. The measurement point for exclusive breastfeeding was at baseline (within 2 days after childbirth), 10th week, 16th week, and 6 months. None of the studies have used the theoretical models guiding the intervention.

### Risk of bias of individual studies

**Fig. 2:**
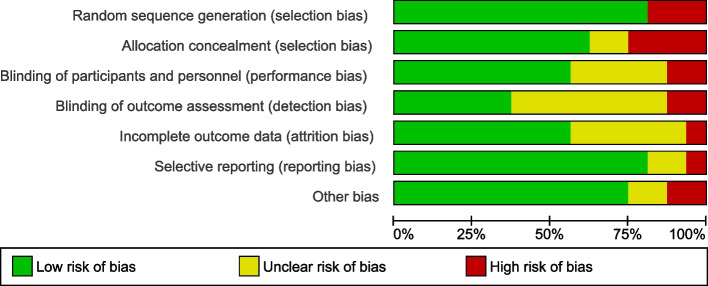
Risk of bias graph of included studies

**Fig. 3:**
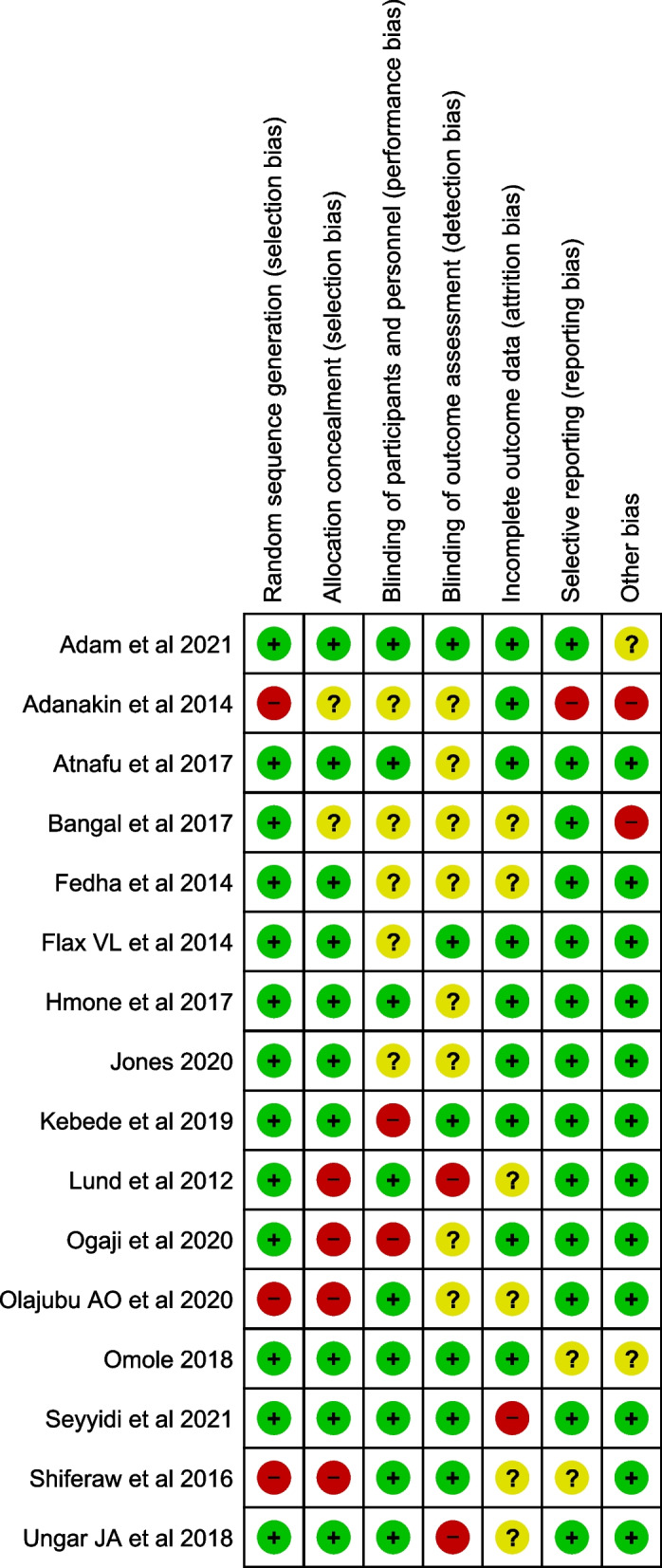
Risk of bias summary of included studies

The overall qualities of the studies included were moderate. Except for two studies, the random sequence generations of included studies were low-risk bias. However, nearly one-third of studies were prone to selection bias because of the non-concealment of the allocation of participants to intervention and control groups. The majority of included studies has a high-risk bias or did not indicate blinding of the outcome assessment (detection bias) within their studies. In more than half of included studies, attrition bias was not presented. The risk of bias graph, and summary were shown in Figs. 2 and 3 respectively. Finally the overall quality of evidence of the current review and meta-analysis was evaluated by the GRADE recommendations [41].

### Study outcome

The primary outcome of the review was to evaluate the effects of mHealth intervention on the level of institutional delivery and postnatal care service utilization among women in low-and lower-middle-income countries. The secondary outcome was to assess the impact of mHealth interventions on the level of exclusive breastfeeding and knowledge of maternal and newborn danger signs among this population in low-and lower-middle-income countries.

### Institutional delivery outcome

**Fig. 4:**
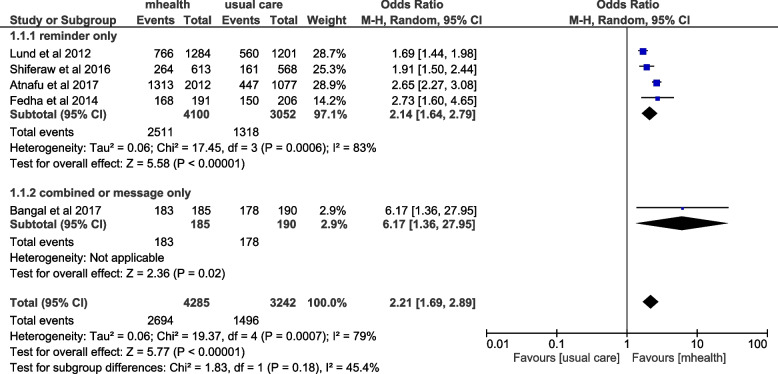
Forest plot of included studies to assess the effect of mhealth intervention on institutional delivery

From the included studies, seven studies have examined the effect of mHealth interventions on the institutional delivery outcome. Among these, six of them reported a significant effect of mHealth while one study has reported that mHealth had no significant effect on institutional delivery. A meta-analysis was done for five of the included articles and the result showed that institutional delivery among women who received mHealth intervention had increased by 121% (OR = 2.21 (95%CI: 1.69–2.89)) compared with women who were only receiving the usual care. The I^2^ statistics show that there was significant heterogeneity among studies (I^2^ = 79%, *p* < 0.001), and thus random-effects model was used. The sensitivity analysis using the one-leave-out approach revealed a trivial difference in the odds of intervention ranging from 1.83 to 2.45, affected by a study conducted by Atnafu et al., 2017. The subgroup analysis based on the intervention characteristics has shown that there was no significant subgroup difference between intervention and control groups based on the intervention characteristics on the outcome of institutional delivery outcome (*P* = 0.17) (Fig. 4).

### Postnatal care outcomes and knowledge of danger signs

**Fig. 5:**
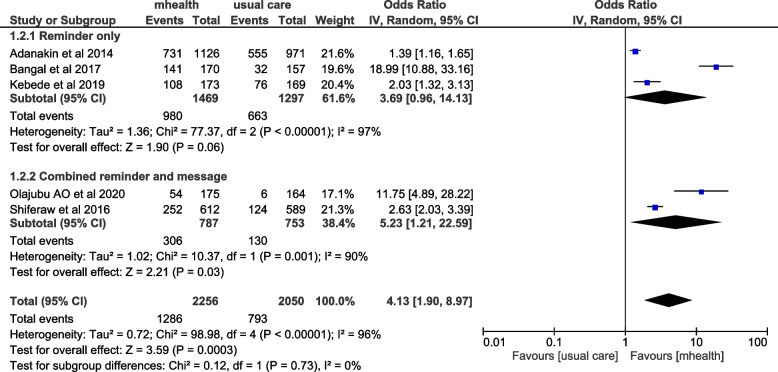
Forest plot of included studies to assess the effect of mhealth intervention on postnatal care uptake

From the included studies, six articles have examined the effect of mHealth intervention on the postnatal care uptake of delivered mothers. Among these, five of them were included in the meta-analysis while one study was not included due to difficulty in finding the figures in the study. Five of the included articles showed that mHealth had significantly improved the odds of uptake of postnatal care services among the intervention group in comparison to the control group. The meta-analysis of these studies revealed that the odds of women who had received phone-based educational messages or reminders were four times more likely to attend full postnatal care visits compared to women who were receiving the usual care (OR = 4.13 (95%CI: 1.90–8.97)). The I^2^ statistics show that there was significant heterogeneity among studies (I^2^ = 96%, *p* < 0.001), and thus random-effects model was used. The sensitivity analysis using the one-leave-out approach revealed an important difference in the odds of intervention ranging from 2.65 to 5.64, which was contributed by two studies conducted by Bangal et al., 2017 and Adanakil et al., 2014. The subgroup analysis based on the intervention characteristics has shown that there was no significant subgroup difference between intervention and control groups based on the intervention characteristics on the outcome of postnatal care utilization (*P* = 0.72) (Fig. 5). One of the studies which were not included in the meta-analysis similarly showed that mHealth intervention has significantly improved the postnatal care-seeking behavior and knowledge of the obstetric danger signs among women who had received both the usual care and mHealth educational message [14].

### Exclusive breast feeding outcomes

**Fig. 6:**
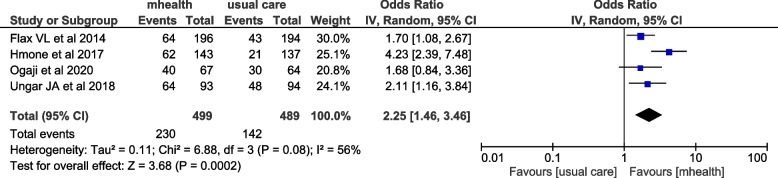
Forest plot of included studies to assess the effect of mhealth intervention on exclusive breast feeding

From the included studies, six articles have examined the effect of mHealth intervention on the level of exclusive breastfeeding. Among these, four articles were included in the meta-analysis. The overall effect of the meta-analysis showed that exclusive breast feeding among women who received mHealth intervention had increased by 125% (OR = 2.25, (95%CI: 1.46–3.46)). The I^2^ statistics showed that there was moderate heterogeneity among studies (I^2^ = 56%, *P* = 0.08) and thus random-effects model was used (Fig. 6). Similarly, a study conducted by Seyyedi et al. showed that the smartphone-based app educational message had a significantly positive effect on breastfeeding self-efficacy and maternal knowledge on exclusive breastfeeding. On the other hand, a study conducted by Adam et al. revealed that mHealth has no significant effect on level of exclusive breastfeeding.

### Publication bias

**Fig. 7:**
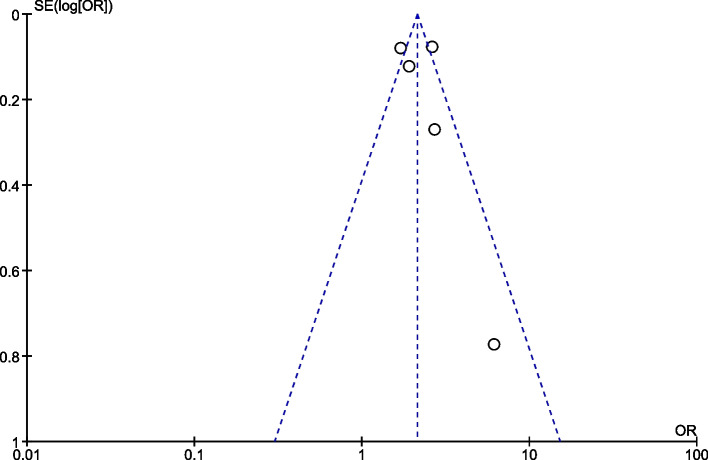
The funnel plot of included studies reporting mhealth intervention on outcome of institutional delivery

**Fig. 8:**
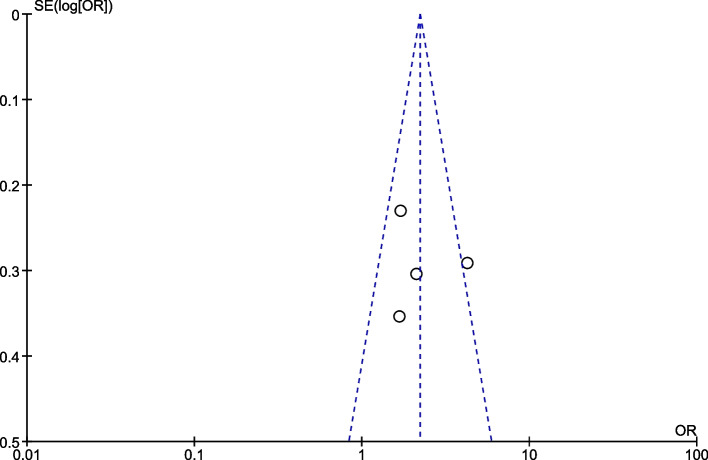
The funnel plot of included studies reporting mhealth intervention on outcome of exclusive breast feeding

**Fig. 9:**
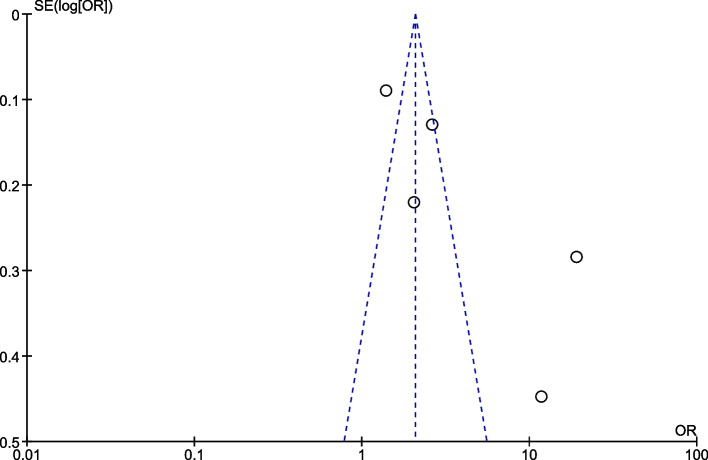
The funnel plot of included studies reporting mhealth intervention on outcome of postnatal care service uptake

The publication bias among included studies was assessed by funnel plot. The symmetry of the funnel plot showed that there was no publication bias for institutional delivery (Fig. 7) and exclusive breastfeeding outcomes (Fig. 8). However, the funnel plot for postnatal care outcome is asymmetrical showing the presence of publication bias (Fig. 9).

## Discussion

This systematic review and meta-analysis was intended to investigate the effect of mHealth interventions on improving facility delivery, postnatal care service utilization, exclusive breastfeeding, and knowledge of obstetric danger signs after childbirth among women in low and lower-middle-income countries. In recent days, the use of mobile technology for the improvement of access to healthcare information and behavior change communication is increasing [42]. In low-income countries, where access to health information is relatively trivial, mobile health communication is supposed to improve the mortality and morbidity of mothers and children. However, the strength of the effect of mHealth intervention, duration, and content of intervention in improving institutional delivery, postnatal care, and related outcomes was not systematically analyzed. Thus, this study aimed to systematically analyze the impact of mHealth intervention in improving the maternal continuum of care particularly facility delivery, postnatal care, and exclusive breastfeeding.

The current meta-analysis showed that women who received educational messages or reminder messages were more likely to give childbirth at a health institution and attended by skilled birth personnel when compared to women who received routine care alone. A similar finding was reported in a review conducted to evaluate the effects of health on antenatal care attendance and facility delivery among pregnant women in low and middle-income countries [43]. A review by Rahman et al., 2022 also revealed that SMS educational message has improved the rate of antennal care and facility delivery despite the fact that the effect is low and needs more investigation [10]. A study conducted in developed countries like Canada and Argentina similarly showed that mhealth had increased the odds of facility delivery, postnatal care uptake and parental self-efficacy [44, 45]. This could be because access to health care information had improved the women’s knowledge and could have influenced their behavior to seek skilled birth by health care personnel.

In this review, the effect of mobile health on the utilization of postnatal care and the improvement of women’s knowledge of obstetric danger signs was also analyzed. The finding of the meta-regression has shown that women who received routine care and phone-based educational message were more likely to adhere to the WHO-recommended postnatal care indications compared to those who only received the usual care. This finding is consistent with a review finding by Mbuthia et al. in that mobile health intervention has improved women’s self-efficacy with demonstrated capacity to adhere to recommended PNC visits, demonstrated ability to recognize and report danger signs, and enhanced capability to exclusively breast their newborns [46]. A similar finding was observed in Canada in which supportive educational program delivered by mHealth program to improve postpartum parental outcomes [45]. In the current study, the knowledge of danger signs among women in the intervention group was significantly better than those women who were receiving only the routine maternal continuum of care [14]. Contrary to this, a study conducted by Adam et al. [12] revealed that mHealth had no significant impact on knowledge of danger signs among women. This could be attributed to the sociocultural differences between respondents between studies. Moreover, it is essential to conduct further studies in this regard to come up with better evidence.

The impact of the mobile educational message on the enhancement of exclusive breastfeeding was systematically analyzed in the current study as well. Four studies were included in the meta-analysis and its pooled effect has shown that SMS educational message has significantly improved the rate of exclusive breastfeeding. This finding is consistent with another review and meta-analysis such that mobile-based interventions had significantly improved the rate of postpartum exclusive breastfeeding, attitude, and efficacy of breastfeeding among women, and reduced health problems in newborns [47, 48]. A study conducted by Seyyedi et al. also showed that the smartphone-based app educational message had a significantly positive effect on breastfeeding self-efficacy and maternal knowledge of exclusive breastfeeding. This might be because mHealth intervention might have enhanced their awareness of exclusive breastfeeding practices and built their trust in intervention providers. In the current review, the majority of included articles have used SMS educational messages and reminders for intervention to convey health care information besides the usual care for subjects in the intervention group. Only four studies used sole reminder messages as a mHealth intervention. The subgroup analysis based on the intervention characteristics showed that there was no significant difference in the effect of mHealth intervention on maternal service outcomes. The result of the finding showed that reminder messages, specific educational messages and combination of both have positively influenced the healthcare service uptake.

## Implications of the findings

The current review has tried to examine the effect of mHealth intervention on the improvement of the maternal continuum of care particularly institutional delivery and postnatal care and related outcomes among women in low-and lower-middle-income countries. However, as available evidence is limited to a few countries, more research should be conducted to reach a definitive conclusion. Thus, this review could help future researchers in giving better insight into the effect of modern mobile technologies on health communications, especially in low-income settings.

## Conclusion

In this meta-analysis, mobile health intervention was found to be effective in improving health-care utilization during childbirth and the postpartum period. Though the interpretation of this review requires caution due to the small number of studies included, the results show that mHealth has the potential to improve health communication among pregnant and laboring women by assisting them in making informed decisions and seeking health care uptake during the critical periods of childbirth and postpartum.

## Recommendations

The finding of the current review and meta-analysis showed that mHealth has a significant effect on improving facility delivery, postnatal care uptake, and rate of exclusive breastfeeding. However, some studies reported inconclusive findings on the effect of mHealth on these outcomes. Thus, we recommend further studies on the impact of mHealth interventions uptake of the maternal and child health care services guided by theoretical frameworks especially focusing its effect on enhancing knowledge of women on obstetric danger signs, ability to report complications, and self-efficacy of women in utilizing services including exclusive breastfeeding.

## Data Availability

The data and materials used for this study were presented within the manuscript.

## Abbreviations

OR: Odds Ratio
PDA: Personal digital assistant
PNC: Postnatal Care
RCT: Randomized controlled trial
SDGs: Sustainable development goals
WHO: World Health Organization
